# Genetic divergence for adaptability and stability in sugarcane: Proposal for a more accurate evaluation

**DOI:** 10.1371/journal.pone.0254413

**Published:** 2021-07-15

**Authors:** João de Andrade Dutra Filho, Tercilio Calsa Júnior, Djalma Euzébio Simões Neto, Lauter Silva Souto, Anielson dos Santos Souza, Rômulo Gil de Luna, Frank Gomes-Silva, Guilherme Rocha Moreira, Moacyr Cunha-Filho, André Luiz Pinto dos Santos, Cícero Carlos Ramos de Brito, Fabiana Aparecida Cavalcante Silva, Andréa Chaves Fiuza Porto, Maria Lindomárcia Leonardo da Costa

**Affiliations:** 1 Biological Sciences Nucleus, Federal University of Pernambuco, Vitória de Santo Antão, Pernambuco, Brazil; 2 Genetics Department, Federal University of Pernambuco, Recife, Pernambuco, Brazil; 3 Carpina Sugarcane Experimental Station, Federal Rural University of Pernambuco, Carpina, Pernambuco, Brazil; 4 Agrarian Sciences Academic Unit, Federal University of Campina Grande, Pombal, Paraíba, Brazil; 5 Environmental Technology Sciences Academic Unit, Federal University of Campina Grande, Pombal, Paraíba, Brazil; 6 Statistics and Informatics Department, Federal Rural University of Pernambuco, Recife, Pernambuco, Brazil; 7 Federal Institute of Pernambuco, Recife, Pernambuco, Brazil; 8 Phytosanitary Diagnosis Laboratory, Northeast Strategic Technologies Center, Recife, Pernambuco, Brazil; 9 Agricultural College Dom Agostinho Ikas, Federal Rural University of Pernambuco, Carpina, Pernambuco, Brazil; 10 Animal Science Department, Federal University of Paraíba, Areia, Paraíba, Brazil; Federal University of Mato Grosso do Sul, BRAZIL

## Abstract

The best agro-industrial performance presented by a crop genotype in one environment may not be reproduced in another owing to complex edaphoclimatic variations. Therefore, breeding programs are constantly attempting to obtain, through artificial hybridization, novel genotypes with high adaptability and stability potential. The objective of this study was to analyze genetic divergence in sugarcane based on the genotypic values of adaptability and stability. A total of 11 sugarcane genotypes were analyzed for eight agro-industrial traits. The genotypic values of the traits were determined using mixed model methodology, and the genetic divergence based on phenotypic and genotypic values was measured using the Mahalanobis distance. The distance matrices were correlated using the Mantel test, and the genotypes were grouped using the Tocher method. Genetic divergence is more accurate when based on genotypic values free of genotype–environment interactions and will differ from genetic divergence based on phenotypic data, changing the genotype allocations in the groups. The above methodology can be applied to assess genetic divergence to obtain novel sugarcane genotypes with higher productivity that are adapted to intensive agricultural systems using diverse technologies. This methodology can also be tested in other crops to increase accuracy in selecting the parents to be crossed.

## Introduction

In sugarcane breeding, the genetic variability available for selection of novel genotypes in families with greater heterotic effects is typically obtained based on preferential crosses between contrasting parents, the constituent genes of which contribute to traits of agro-industrial interest [1]. As sugarcane is an allogamous species and sensitive to inbreeding depression, crossing between related individuals should be avoided [2].

The emergence of dissimilarity analyses along with multivariate techniques has made it possible to perform crossing based on considerations of the genetic divergence between genotypes, even under circumstances in which the divergence is based on quantitative traits that are often influenced to a considerable extent by environmental factors. In this context, several studies have been conducted on the genetic divergence of a diverse range of crops, including opium poppy [3], eucalyptus [4], passion fruit [5], tomato [6], beans [7], and sweet sorghum [8], for which generalized Mahalanobis distance models have been used that take into consideration the residual variances and covariances that exist in the quantitative traits of the examined genotypes [9].

In the case of sugarcane, in addition to considering agro-industrial traits such as tons of sugarcane per hectare (TCH), tons of pol per hectare (TPH), and total recoverable sugar (TRS), it is essential that genetic divergence be based on the adaptability and stability of the parents, with the objective of obtaining families, and consequently superior genotypes, adaptable to a range of different cultivation environments.

With respect to genotype performance, the term adaptability refers to the ability of a given genotype to respond advantageously to the prevailing environmental conditions, whereas stability indicates the limited extent of variation in performance when exposed to different environments [10]. In field experiments, although measurements of traits such as diameter, height, and stalk number are reasonably straightforward, it is considerably more difficult to simultaneously determine adaptability and stability for analyses of genetic divergence. There are, nevertheless, currently several methodological approaches available for this type of analysis, including those based on the analysis of variance (ANOVA) proposed by Annichiarico [11], non-parametric analysis proposed by Lin and Bins [12], and regression analysis proposed by Eberhart and Russel [13].

The parameters of adaptability and stability considered when using such methodologies are based on the performance of genotypes (expressed in terms of quantitative traits) evaluated in several environments. However, Cruz *et al*. [9] stated that the minimum variance shown by genotypes in different environments represents only phenotypic stability. When any of these classical methodologies are used, environmental variance is present in the various traits used to determine phenotypic adaptability and stability parameters [14]. The respective environmental and genetic variations in these phenotypic traits are used in the analysis of genetic divergence.

The advent of mixed linear models has made it possible to undertake simultaneous selection for productivity, adaptability, and stability based on the harmonic mean of the relative performance of genotypic values (HMRPGVs) among groups of genotypes evaluated in different environments [15]. In other words, for each quantitative trait evaluated, there is a parameter of adaptability and stability for genotypes in multiple environments. These HMRPGVs can be used to analyze genetic divergence, and the results obtained tend to be more accurate with respect to recommendations for the crosses to be performed. This is because mixed models yield results that can be presented in terms of the unit or scale of the evaluated trait, which can be interpreted as genotypic values, a property that is generally not available when using other methodologies [16].

Using mixed linear models, Lopes *et al*. [17] evaluated the genetic divergence of 138 sugarcane genotypes in three environments, although not all these genotypes used as common treatment were examined in all three environments. However, Zeni-Neto *et al*. [18] indicated that the HMRPGV approach should be employed with caution when selecting for stability and productivity in unbalanced experiments in which not all genotypes are present in all assessed environments.

Nevertheless, in balanced experiments in which all genotypes are present in the assessed environments and evaluations are conducted over more than one harvest cycle, linear mixed models can be effectively adopted to obtain robust and reliable results.

In this way, genetic divergence will be more accurate if based on genotypic values free of genotype–environment interaction and will differ from genetic divergence based on phenotypic data and may even change the genotype allocations in the groups, encouraging the reassessment of crossing planning.

In this study, we used the HMRGPV approach with the genetic values already discounted from instability and capitalized by adaptability, that is, the HMRPGV × overall mean in all the environments (GM), in the analysis of genetic divergence in sugarcane.

## Material and methods

### Plant material and evaluated environments

In this study, five experiments over a 2-year period (2010–2011) were conducted wherein the performance of sugarcane plant and ratoon cane crops in the five following sugar mills was examined: Sugar mills União e Indústria, Cucaú, Olho d’Água, Petribú, and Santa Tereza. According to the classification proposed by Koffler *et al*. [19], these sugar mills represent respectively the sugarcane microregions of the south forest, south coast, north forest, central region, and north coast of the state of Pernambuco, Brazil (Table 1).

**Table 1:** Environment description, geographical coordinates of assessed locations, and general data on experimental conditions.

Cities	Env	Altitude m	Latitude ^0^S	Longitude ^0^W	Harvest cycle	Planting date	Harvest date	RI mm
Primavera	South Forest	129	08°19’53"	35°21’15"	Plant	10/08/09	12/08/10	1500
					Ratoon	12/08/10	14/08/11	1750
Rio Formoso	South Coast	5	8°39’50"	35°09’32"	Plant	18/08/09	20/08/10	2000
					Ratoon	20/08/10	22/08/11	3000
Camutanga	North Forest	98	07°24’25"	35°16’28"	Plant	28/08/09	30/08/10	1100
					Ratoon	30/08/10	30/08/11	1400
Lagoa do Itagenga	Central Region	183	07°56’10"	35°17’25"	Plant	03/09/09	05/09/10	1200
					Ratoon	05/09/10	08/09/11	1400
Goiana	North Coast	13	07°33’38"	35°00’ 09"	Plant	10/09/09	12/09/10	1300
					Ratoon	12/09/10	14/09/11	2200

Env, environment; RI, rainfall index.

For each experiment, the performance of 11 genotypes was assessed. Eight of these genotypes were promising clones of the RB01 series undergoing the final phases of trials conducted by the Sugarcane Genetic Improvement Program of the Universidade Federal Rural de Pernambuco, a member of the Interuniversity Network for the Development of the Sugar and Energy Sector (PMGCA/UFRPE/RIDESA). The remaining three genotypes were the standard cultivars RB833129, RB92579, and SP79-1011 (Table 2).

**Table 2:** Sugarcane clones of the RB01 series and standard cultivars used in the analysis of genetic divergence.

Genotypes	Parents	Breeder institution
1. RB012633	RB934530 × SP81-3250	UFRPE
2. RB012650	RB75126 × SP81-3250	UFRPE
3. RB012688	SP79-2313 ×?	UFRPE
4. RB012693	BJ7504 ×?	UFRPE
5. RB012726	RB93153 × F150	UFRPE
6. RB012776	RB931555 × RB83160	UFRPE
7. RB012777	RB72454 × RB831970	UFRPE
8. RB012801	SP82-3530 × RB83160	UFRPE
9. RB863129*	RB763411 ×?	UFRPE
10. RB92579*	RB75126 × RB72199	UFAL
11. SP79-1011*	NA56-79 × CO775	COPERSUCAR

* Standard cultivars. UFRPE, Federal Rural University of Pernambuco; UFAL, Federal University of Alagoas; COPERSUCAR, Brazilian Sugar and Ethanol Cooperative.

### Experimental design and cultural practices

For each of the assessed environments and production systems, experiments were arranged in a randomized complete block design with four replications (blocks). Planting dates varied according to the respective environments and production systems within each environment (Table 1). Each experimental unit (genotype) consisted of five 8-m-long rows spaced 1 m apart. In each row, there were 10 plants (reeds) per meter for a total of 400 plants in each experimental unit. Soil pH correction and fertilization in plots were carried out in accordance with the respective agro-industrial company production systems.

### Data collection

In each environment, the crops were harvested 12 months after planting (Table 1). At the time of harvest, the following traits were evaluated: tons of pol per hectare (TPH/t·ha^-1^), tons of sugarcane per hectare (TCH/t·ha^-1^), fiber (FIB%), corrected pol% (CP%), purity (PT%), soluble solids content (SSC%), total recoverable sugar (TRS/kg), and tons of total recoverable sugar per hectare (TRS/t·ha^-1^).

Values for the traits TCH, TPH, and TRS were estimated using the following formulas:

$$\text{TCH}=\text{Total}\;\text{weight}\;\text{of}\;\text{plot}\times 10/\text{usable}\;\text{area}\;\text{of}\;\text{plot}\;\text{in}\;{\text{m}}^{2}$$ (1)

TPH=TCH×CP/100 (2)

TRS:TCH×TRS/1000 (3)

SSC was measured using a field refractometer, with readings obtained from homogeneous samples of a broth prepared from 10 stalks collected at random from each plot. To calculate the fiber traits CP, PT, and TRS, the methodology proposed by Fernandes was adopted [20].

### Statistical analysis

Three-way ANOVA was performed according to the following statistical model presented by Cruz *et al*. [9]:

$$Y_{ijkm}=\mu +G_{i}+Y_{j}+E_{k}+(\frac{B}{Y})/E_{jkm}+{GY}_{ij}+{GE}_{ik}+{YE}_{jk}+{GYE}_{ijk}+{Ɛ}_{ijkm}$$

In which:

i = 1, 2,…, g

j = 1, 2,…, y

k = 1, 2,…, e

m = 1, 2,…, r,

where μ is the overall mean;

Gi, Yj, and Ek are the effects of genotypes, years, and environments, respectively;

GYij, GEik, and YEjk are the effects of first-order interactions between genotypes and years, genotypes and environments, and years and environments, respectively;

GYEijk is the effect of the triple interaction among genotypes, years, and environments;

(B/Y)/Ejkm is the effect of blocks within years within environments and;

Ɛijkm is random error.

Prior to conducting the three-way ANOVA, the Hartley test was applied to identify the homogeneity of residual variances. The genetic parameters genotypic variance, variance of genotype × year interactions, variance of genotype × environment interactions, variance of genotype × environment × year interactions, mean heritability, coefficient of genetic variation, and index b were estimated according to the methods reported by Cruz *et al*. [9]. When significant effects were detected, separation of means was performed to examine the differences between genotypes using Scott and Knott grouping [21] at the 5% level of probability. Analysis of gross economic rentability performed to select the most productive genotypes was based on the formula:

$$\text{TRS}\;\text{kilo}\;\text{price}\times \text{TRS}\;\left(\text{kg}/\text{t}\right)\times \text{TCH}$$ (4)

Genetic divergence among the evaluated genotypes based on phenotypic and genotypic data was determined by employing a measure of dissimilarity, the generalized distance of Mahalanobis, using the following equation:

$$D_{ii'}^{2}=\delta^{'}\psi^{-1}\delta$$ (5)

Where

$D_{ii^{'}}^{2}$ is the distance of Mahalanobis dissimilarity between genotypes i and i’;

***ψ*** is the matrix of residual variances and covariances (dimension 11);

δ=[d1d2…dv] (6)

dj=Yij-Yi'j (7)

where dv represents the difference between the means of two genotypes i and i’ for a specific trait j, and Yij is the mean of the i^th^ genotype in relation to the j^th^ variable.

For joint deviance analysis (ANADEV), the following model was used:

$$y=X_{f}+Z_{g}+Q_{a}+T_{i}+W_{t}+e$$

where y is the data vector;

f is the vector of the effects of the repetition–environment–year combinations (assumed to be fixed) added to the overall mean;

g is the vector of genotypic effects (assumed to be random);

a is a vector of the effects of the interaction of genotypes with years (random);

i is the vector of the effects of genotype × environment interactions;

t is the vector of the effects of triple genotypes × environment × year interactions (assumed to be random); and

e is the vector of errors or residuals (random). Uppercase letters represent the incidence matrices for the referred effects.

Using this model, we obtained the components of variance and predictors: Restricted Maximum Likelihood and Best unbiased linear predictor (REML/BLUP) given by $\hat{\mu }j+{\hat{g}}_{i}$, where $\hat{\mu }j$ is the mean of environment j and ${\hat{g}}_{i}$ is the prediction of the genotypic effect.

The prediction of genotypic values, which takes into consideration the mean interaction (gem) in the different environments, was obtained using the model $\hat{\mu }j+\hat{g}i+\hat{g}em$, calculated using the following equation:

$$\hat{\mu }\left\{\left[\frac{{\hat{\sigma }}_{g}^{2}+{\hat{\sigma }}_{c}^{2}}{n}\right]{\hat{\sigma }}_{g}^{2}\right\}{\hat{g}}_{i}$$ (8)

Where:

μ is the overall mean of all environments;

n is the number of environments; and

${\hat{g}}_{i}$ is the specific genotypic effect (genotype i).

Joint selection, which considers the average productivity, stability, and adaptability of genotypes, was determined based on the HMRPGV using the following equation:

$$HMRPGVi=n/{(Ʃ}_{j=1}^{n}1/Vgij)$$ (9)

Where:

n is the number of environments where genotype i was evaluated, and

Vgij is the genotypic value of genotype i in environment j expressed as the average proportion of this environment.

The HMRPGV values were multiplied by the GM, which resulted in the same order of magnitude of the evaluated trait.

Dissimilarity matrices based on phenotypic and genotypic values were correlated using the Mantel matrix comparison test, with 1,000 simulations to verify the level of agreement and divergence between the results [9].

The grouping of genotypes, based on phenotypic and genotypic values of adaptability and stability, was carried out using the Tocher optimization method. All statistical analyses of genetic parameters were performed using Genes [22] and Selegen [23] software.

## Results

### Three-way ANOVA and Scott–Knot grouping

ANOVA revealed significant differences between genotypes for all assessed traits evaluated in the five sugarcane microregions in the state of Pernambuco. The analyses also revealed significant differences between the two assessed years, environments, and the genotype × year (harvest cycles), genotype × environment, and genotype × year × environment interactions. The obtained coefficient of variation values ranged from low to medium, indicating excellent experimental precision. Hartley’s test, in turn, indicated homogeneity of the residual variances, thereby revealing the accuracy of the respective ANOVA (Table 3).

**Table 3:** Summary of joint analysis of variance of the traits of sugarcane genotypes evaluated over two consecutive years during the final phases of trials conducted in sugarcane microregions in the State of Pernambuco.

		Mean squares							
SV	DF	TPH (t.ha^-1^)	TCH (t.ha^-1^)	FIB (%)	CP (%)	PT (%)	SSC (%)	TRS (kg)	TRS/(t.ha^-1^)
**Genotypes**	10	128.9**	5430**	7.22**	3.89*	48.2*	2.1*	189*	121**
**Years**	1	1362.2*	62785*	3.04^ns^	1.24^ns^	174^ns^	1.5^ns^	0.01^ns^	1348*
**Environments**	4	946.4**	36567**	53.2**	171**	813**	361**	13660**	846**
**G × Y**	10	8.7*	308.9*	1.13^ns^	1.37^ns^	5.9^ns^	2.2*	96^ns^	8.2*
**G × E**	40	13.49**	519**	2.12**	1.70**	22.3**	1.6**	96**	12.3**
**Y × E**	4	189.1**	6329**	17.5**	36.8**	78**	117**	3426**	188**
**G × Y × E**	40	3.79**	124.9*	1.15*	0.76^ns^	10.7*	1.0^ns^	51^ns^	3.46**
**Residue**	300	2.07	79.63	0.72	0.68	7.10	0.84	45	1.88
**Mean**		10.19	68.19	14.4	14.8	86.4	21.2	145.7	9.98
**CVe (%)**		14.14	13.08	5.89	5.56	3.0	4.32	4.6	13.7
**H**		4.0	4.4	4.20	3.0	7.0	2.9	2.7	3.7

**p = 0.05 and *p = 0.01 determined using an F test. ns, not significant; SV, source of variation; DF, degrees of freedom; G × Y, genotype × year interaction; G × E, genotype × environment interaction; Y × E, years × environment interaction; G × Y × E, genotype × year × environment interaction; CVe, coefficient of variation; H, Hartley test; TCH, tons of cane per hectare; TPH, tons of pol per hectare; FIB, fiber; CP, corrected pol%; PT, purity; SSC, soluble solids content; TRS, total recoverable sugars; and TRS/t·ha^-1^, total recoverable sugars per hectare.

Three trait-related groups based on Scott–Knott grouping were obtained, namely, TPH (t.ha^-1^), TCH (t.ha^-1^), and TRS (t.ha^-1^), which singled out genotype RB92579, allocated to group a, given that it showed the highest productivity and gross economic profitability among the assessed genotypes. The genotypes RB012777, RB863129, and SP79-1011 were also noteworthy, with estimated values above the average for these three traits, and were therefore allocated to group b. The other genotypes, with values below the average, were allocated to group c (Table 4).

**Table 4:** Grouping of averages of the traits of sugarcane genotypes evaluated over two consecutive years during the final phases of trials conducted in sugarcane microregions in the State of Pernambuco.

	Traits								
Genotypes	TPH (t.ha^-1^)	TCH (t.ha^-1^)	FIB (%)	CP (%)	PT (%)	SSC (%)	TRS (kg)	TRS (t.ha^-1^)	E_R_ (R$.ha^-1^)
**RB92579**	13.9a	92.4a	14.1a	15.1a	87.4a	21.2a	147.7a	13.5a	10.917,9
**RB012777**	11.7b	75.8b	14.5a	15.5a	88.3a	21.6a	150.0a	11.3b	9.096,00
**RB863129**	11.7b	80.1b	13.8a	14.3a	84.3a	20.7a	145.4a	11.5b	9.317,23
**SP79-1011**	10.7b	72.0b	14.0a	14.8a	86.9a	21.9a	145.4a	10.5b	8.375,04
**RB012726**	10.2c	67.9c	14.3a	15.0a	87.0a	21.2a	147.0a	9.9c	7.985,04
**RB012650**	10.1c	68.6c	14.7a	14.6a	86.0a	21.1a	143.8a	9.9c	7.891,44
**RB012688**	9.6c	64.2c	14.3a	14.8a	85.8a	21.3a	145.7a	9.4c	7.483,15
**RB012033**	9.2c	61.9c	14.0a	15.0a	86.2a	21.3a	147.2a	9.1c	7.289,34
**RB012801**	8.8c	59.7c	15.2a	14.7a	86.1a	21.4a	144.5a	8.6c	6.901,32
**RB012693**	8.0c	54.5c	14.9a	14.8a	87.1a	21.2a	144.9a	7.9c	6.317,64
**RB012776**	7.6c	52.6c	14.5a	14.5a	85.3a	21.1a	143.7a	7.5c	6.046,89

Means followed by the same letter in columns belong to the same group based on Scott–Knott grouping at p = 0.05. Er, economic return; R$·ha^-1^, Reais per hectare; TCH, tons of cane per hectare; TPH, tons of pol per hectare; FIB, fiber; CP, corrected pol%; PT, purity; SSC, soluble solids content; TRS, total recoverable sugars; and TRS/t·ha^-1^, total recoverable sugars per hectare.

### Estimation of genetic parameters

With respect to genetic parameters, we detected genotypic variance superior to residual variance and variances of interactions for the traits TPH, TCH, and TRS (t.ha^-1^). Heritability coefficients showed high magnitude for the traits TPH, TCH, and TRS (t.ha^-1^) (> 75) and average magnitude for the traits FIB, CP, PT, and TRS (kg) (> 50), leading to significant gains in the selection of these materials for the assessed environments. For these traits, the genotypic coefficient of variation was higher than 10, and the ratio between the coefficient of genetic variation and the experimental coefficient of variation was higher than the unit. This indicates the predominance of the genetic component, thereby contributing to effective selection (Table 5).

**Table 5:** Components of variance and genetic parameters of the traits of sugarcane genotypes evaluated over two consecutive years during the final phases of trials conducted in sugarcane microregions in the State of Pernambuco.

	Genetic parameters							
Traits	G _V_	V_G x Y_	V_G x E_	V_G x Y x L_	V _R_	H^2^ (%)	CVg	CVg/CV_E_
**TPH/t.ha**^**-1**^	2.89	0.25	1.30	0.18	2.08	90	16.7	1.17
**TCH/t.ha**^**-1**^	122.8	9.2	49.2	4.52	79.6	90	16.2	1.24
**FIB**	0.13	0.0	0.16	0.04	0.72	71	2.47	0.42
**CP**	0.05	0.03	0.12	0.07	0.68	56	1.57	0.28
**PT**	0.64	0.0	1.72	0.36	7.10	54	0.93	0.30
**SSC**	0.01	0.06	0.08	0.01	0.84	26	0.57	0.13
**TRS**	2.33	2.26	5.78	0.58	45	50	1.04	0.23
**TRS/t.ha**^**-1**^	2.72	0.24	1.18	0.16	1.88	90	16.52	1.20

G_V_, genotypic variance; V_G_ × _Y_, variance of genotype × year interactions; V_G_ × _E_, variance of genotype × environment interactions; V_G_ × _Y_ × _E_, variance of genotype × year x environment interactions; V_R_, residual variance; H^2^, average heritability; CVg, coefficient of genetic variation; CVg/CVe, index b; TCH, tons of cane per hectare; TPH, tons of pol per hectare; FIB, fiber; CP, corrected pol%; PT, purity; SSC, soluble solids content; TRS, total recoverable sugars; and TRS/t·ha^-1^, total recoverable sugars per hectare.

### Estimation of genetic gain and genotypic parameters of adaptability and stability

In all environments, the genotype RB92579 showed the highest genotypic values, the highest predicted average free of the interaction $\left(\hat{\mu }+\hat{g}\right)$, and the highest overall average, followed by genotypes RB012777, RB863129, and SP79-1011, thereby indicating the productive superiority of these materials over those of the other evaluated genotypes (Table 6).

**Table 6:** Estimates of components of predicted averages and genotypic values (individual best unbiased linear predictor) of TCH(t.ha^-1^), TPH(t.ha^-1^), and TRS(t.ha^-1^) of sugarcane genotypes evaluated over two consecutive years during the final phases of trials conducted in sugarcane microregions in the State of Pernambuco.

	Traits				
	TPH (t.ha^-1^)				
Genotypes	Genotypic effect	$\left(\hat{\mu }+\hat{g}\right)$	Genetic gain	Average	$\left(\hat{\mu }+\hat{g}+\hat{g}em\right)$
**RB92579**	3.19	13.38	3.19	13.38	13.61
**RB012777**	1.58	11.76	2.38	12.57	11.83
**RB863129**	1.37	11.56	2.05	12.23	11.66
**SP79-1011**	0.40	10.59	1.63	11.82	10.62
**RB012726**	0.07	10.24	1.32	11.50	10.25
**RB012650**	- 0.16	10.02	1.07	11.26	10.01
**RB012688**	- 0.65	9.53	0.82	11.01	9.48
**RB012033**	- 0.87	9.31	0.61	10.80	9.24
**RB012801**	- 0.93	9.25	0.44	10.62	9.18
**RB012693**	- 1.97	8.20	0.20	10.38	8.06
**RB012776**	- 2.01	8.18	0.00	10.18	8.02
	**TCH (t.ha**^**-1**^**)**				
**RB92579**	21.57	89.77	21.57	89.77	91.11
**RB012777**	8.45	76.65	13.88	82.08	77.18
**RB863129**	11.63	79.82	16.60	84.79	80.55
**SP79-1011**	2.67	70.82	11.08	79.28	71.04
**RB012726**	- 0.65	67.53	7.23	75.42	67.49
**RB012650**	- 0.30	67.89	8.80	77.00	67.87
**RB012688**	-4.85	63.33	5.50	73.69	60.03
**RB012033**	-5.92	62.26	4.07	72.27	61.89
**RB012801**	-6.28	61.91	2.92	71.11	61.51
**RB012693**	-13.20	54.99	0.00	68.19	54.17
**RB012776**	-13.11	55.07	1.32	69.51	54.25
	**TRS (t.ha**^**-1**^**)**				
**RB92579**	3.08	13.06	3.08	13.06	13.28
**RB012777**	1.45	11.43	2.00	11.53	11.53
**RB863129**	1.46	11.44	2.27	12.25	11.55
**SP79-1011**	0.38	10.37	1.59	11.58	10.38
**RB012726**	-0.01	9.96	1.27	11.25	9.96
**RB012650**	-0.13	9.84	1.04	11.02	9.84
**RB012688**	-0.61	9.36	0.80	10.78	9.32
**RB012033**	-0.83	9.14	0.59	10.58	9.08
**RB012801**	-0.92	9.05	0.43	10.41	8.99
**RB012693**	-1.94	0.00	0.00	9.98	7.90
**RB012776**	-1.93	0.19	0.19	10.17	7.91

TCH, tons of sugarcane per hectare; TPH, tons of pol per hectare; TRS, total recoverable sugars.

The parameters of genotypic adaptability and stability (HMRPGV and HMRPGV × GM) are presented in Table 7. The genotype RB92579 was the most stable with respect to the traits TCH, TPH, and TRS (t·ha^-1^), followed by the genotypes RB012777, RB863129, and SP79-1011.

**Table 7:** Adaptability and stability (harmonic mean of the relative performance of genotypic values (HMRPGV) and HMRPGV × overall mean in all the environments) of the genotypic values of sugarcane genotypes with prediction based on analysis of the best unbiased linear predictor evaluated over two consecutive years during the final phases of trials conducted in sugarcane microregions in the State of Pernambuco.

	Traits					
	TPH (t·ha^-1^)		TCH (t·ha^-1^)		FIB	
Genotypes	HMRPGV	HMRPGV × GM	HMRPGV	HMRPGV × GM	HMRPGV	HMRPGV × MGM
**RB92579**	1.35	13.69	1.33	91.19	0.99	14.33
**RB012777**	1.17	11.96	1.13	77.60	1.00	14.51
**RB863129**	1.14	11.65	1.18	80.72	0.97	14.01
**SP79-1011**	1.04	10.60	1.04	71.10	0.99	14.24
**RB012726**	1.00	10.27	0.98	67.48	0.99	14.33
**RB012650**	0.97	9.91	0.99	67.61	1.01	14.62
**RB012688**	0.92	9.32	0.91	62.07	0.98	14.24
**RB012033**	0.91	9.29	0.91	62.26	0.98	14.26
**RB012801**	0.89	9.09	0.90	61.09	1.04	15.05
**RB012693**	0.79	8.00	0.79	53.86	1.01	14.70
**RB012776**	0.75	7.63	0.76	51.52	0.99	14.40
	**CP**		**PT**		**SSC**	
**RB92579**	1.00	15.01	1.01	87.44	1.00	21.22
**RB012777**	1.02	15.21	1.01	87.96	1.00	21.39
**RB863129**	0.97	14.55	0.98	84.51	0.99	21.03
**SP79-1011**	1.00	14.87	1.00	87.17	0.99	21.09
**RB012726**	1.00	15.02	1.00	86.94	1.00	21.26
**RB012650**	0.99	14.75	1.00	86.12	1.00	21.18
**RB012688**	1.00	14.94	1.00	86.36	1.00	21.27
**RB012033**	1.00	14.88	0.99	85.86	1.00	21.26
**RB012801**	0.99	14.82	1.00	86.18	1.00	21.31
**RB012693**	1.00	14.86	1.00	86.75	1.00	21.21
**RB012776**	0.99	14.87	0.99	85.54	1.00	21.18
	**TRS (kg)**			**TRS (t·ha**^**-1**^**)**		
**RB92579**	1.00	146.29		1.34	13.33	
**RB012777**	1.01	147.40		1.16	11.60	
**RB863129**	0.99	144.14		1.16	11.55	
**SP79-1011**	1.00	145.40		1.03	10.37	
**RB012726**	1.00	146.49		1.00	9.98	
**RB012650**	0.99	144.89		0.98	9.75	
**RB012688**	1.00	146.18		0.92	9.15	
**RB012033**	1.00	145.89		0.92	9.16	
**RB012801**	1.00	145.39		0.89	8.92	
**RB012693**	1.00	145.38		0.79	7.84	
**RB012776**	1.00	145.70		0.75	7.54	

HMRPGV, harmonic mean of the relative performance of genotypic values; GM, overall mean in all the environments; TCH, tons of sugarcane per hectare; TPH, tons of pol per hectare; FIB, fiber; CP, corrected pol%; PT, purity; SSC, soluble solids content; TRS, total recoverable sugars; and TRS/t·ha^-1^, total recoverable sugars per hectare.

The genotypes RB012777, RB012650, RB012801, and RB012693 were the most stable with respect to PT and FIB; RB012777, RB92579, SP79-1011, RB012726, RB012688, RB012033, and RB012693 were the most stable with regard to CP; and RB92579, RN012777, SP79-1011, RB012726, RB012650, RB012688, RB012801, and RB012693 were the most stable for PT. All genotypes showed stability for the traits SSC and TRS, with the only exceptions being RB863129 and SP79-1011 for SSC and RB863129 and RB012650 for TRS (kg).

When using mixed models, genotypes showing values equal to or greater than unity are considered comparatively stable. From a genetic perspective, stability is indicative of consistency in the phenotypic expression of different traits evaluated in different environments. The data presented in Table 8 indicate the relatively minor contribution of non-genetic variance in the phenotypic variance of the evaluated traits.

**Table 8:** Variance components (individual REML) of the traits evaluated over two consecutive years during the final phases of trials conducted in sugarcane microregions in the State of Pernambuco.

	TPH (t·ha^-1^)	TCH (t·ha^-1^)	FIB (%)	CP (%)	PT (%)	SSC (%)	TRS (kg)	TRS (t·ha^-1^)
**C**_**2**_**gy**	0.04	0.03	0.00	0.05	0.00	0.04	0.05	0.04
**C**_**2**_**ge**	0.15	0.14	0.11	0.17	0.17	0.15	0.15	0.15
**C**_**2**_**gey**	0.06	0.02	0.14	0.04	0.05	0.09	0.08	0.05
**Rge**	0.73	0.76	0.42	0.13	0.28	0.01	0.03	0.74
**Rgy**	0.90	0.92	0.95	0.33	0.98	0.04	0.09	0.90
**rge_y**	0.73	0.77	0.43	0.31	0.28	0.23	0.27	0.75
**rgy_e**	0.93	0.94	0.98	0.80	0.99	0.77	0.75	0.92
**rge_my**	0.75	0.77	0.58	0.29	0.35	0.32	0.32	0.76
**rgy_me**	0.91	0.93	0.97	0.58	0.99	0.54	0.50	0.91
**Rgey**	0.62	0.68	0.24	0.08	0.23	0.00	0.01	0.63

C2gy, coefficient of determination of the effects of genotype × year interactions; C2ge, coefficient of determination of the effects of genotype × environment interactions; C2gey, coefficients of determination of the effects of genotype × environment × year interactions; rge, genotypic correlation through the environment valid for any year; rgy, genotypic correlation over the two years valid for any environment; rge_y, genotypic correlation through the environment in a given year; rgy_e, genotypic correlation over the two years in a given environment; rge_my, genotypic correlation across environments for the average of all years; rgy_me, genotypic correlation over the years for the average of all environments; rgey, genotypic correlation across environments and years; TCH, tons of sugarcane per hectare; TPH, tons of pol per hectare; FIB, fiber; CP, corrected pol%; PT, purity; SSC, soluble solids content; TRS, total recoverable sugars; and TRS/t·ha^-1^, total recoverable sugars per hectare.

### Analysis of genetic divergence

Table 9 shows the genetic divergence analyses conducted using the generalized Mahalanobis distance for obtaining phenotypic and genotypic results.

**Table 9:** Genetic divergence among 11 sugarcane genotypes based on eight agro-industrial traits evaluated over two consecutive years during the final phases of trials conducted in sugarcane microregions in the State of Pernambuco.

	**Distance between genotypes**										
**G**	1	2	3	4	5	6	7	8	9	10	11
**1**		4.76	**0.67**	4.63	1.15	3.06	4.41	4.22	9.99	14.98	14.99
**2**	1.59		7.10	2.53	7.44	4.74	9.82	2.36	3.20	9.59	5.34
**3**	0.72	**0.50**		7.05	1.83	3.90	5.33	4.88	11.95	18.70	20.41
**4**	1.95	2.88	2.28		8.74	1.38	14.19	2.23	10.90	19.72	9.10
**5**	0.97	0.98	1.05	3.08		7.20	2.01	7.09	11.79	13.25	18.59
**6**	1.76	3.48	2.12	0.75	3.82		13.66	2.29	12.95	**24.18**	14.97
**7**	3.61	2.14	3.21	6.67	1.71	8.73		11.41	11.63	8.16	18.14
**8**	2.27	1.74	1.87	0.73	2.40	1.63	5.16		8.63	18.28	13.37
**9**	5.43	3.42	4.25	11.30	4.36	10.62	4.04	9.31		5.64	7.17
**10**	12.19	8.37	11.31	19.69	8.36	**21.63**	4.16	16.49	4.34		8.73
**11**	2.05	1.15	1.47	5.31	1.40	5.72	1.51	4.67	1.83	6.16	

Values above the diagonal are the dissimilarity measures obtained based on the parameters of adaptability and genotypic stability (harmonic mean of the relative performance of genotypic values × overall mean in all the environments) and those below the diagonal are dissimilarity measures obtained from phenotypic data. G, genotypes.

**Fig 1:**
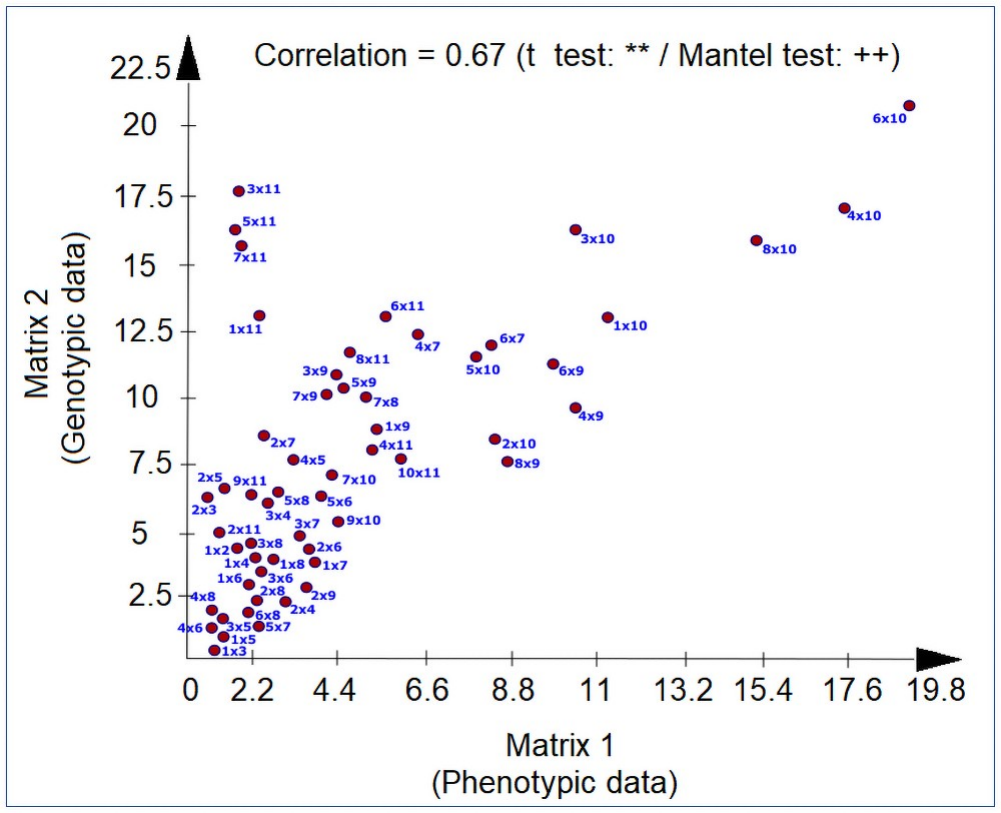
Graphic dispersion of pairs of sugarcane genotypes determined based on dissimilarity measurements obtained from phenotypic (x axis) and genotypic (y axis) data. The correlation between the matrices is illustrated. **p = 0.01, as determined using a *t* test; ^++^p = 0.01, as determined using the Mantel test based on 1,000 simulations.

With regard to Mahalanobis distance based on the phenotypic data, we established that the greatest distance was that between genotypes RB012776 and RB92579 (D^2^ = 21.63), whereas the genotypes RB0122650 and RB012688 showed the smallest distance (D^2^ = 0.50). Consistently, genotypes RB012776 and RB92579 showed the greatest distance (D^2^ = 24.18) with respect to the Mahalanobis distance based on the genotypic data for adaptability and stability, although in this case, the smallest distance was detected between genotypes RB012688 and RB012633 (D^2^ = 0.67). Moreover, there was a moderate correlation between the dissimilarity matrices, as indicated by the coefficient value of 0. 67 obtained using the Mantel test, as illustrated in Fig 1.

This moderate correlation between the dissimilarity matrices showed differences in the estimate of genetic divergence in the evaluated genotypes. The genetic divergence based on genotypic data changed the genotype allocations in the groups (Table 10).

**Table 10:** Genotype groups established using the Tocher method based on generalized mahalanobis distances (phenotypic and genotypic data).

Groups	Phenotypic data	Genotypic data
**I**	2, 3, 5, 1, 11, 7, 8, 4, and 6	1, 3, 5, and 7
**II**	9	2, 4, 6, and 8
**III**	10	9 and 10
**IV**	-	11
**Intergroup boundary (θ)**	4.17	5.65

These results are of fundamental importance with respect to the planning of trait improvement programs designed to identify novel genotypes with greater heterotic effects, as these provide an indication of the progenitors of distinct groups or subgroups that ideally should be included in crossings.

## Discussion

Significant differences between genotypes, detected using ANOVA, provide an indication of variations in the phenotypic expression of evaluated traits that are considered the most important components of sugarcane productivity, and can be attributed to the different alleles that comprise the genetic makeup of the respective genotypes. The importance of biological variation has been highlighted in many studies, including by Mougi [24], who stated that it is an essential element for the operation of natural selection. Given that genetic improvement is essentially an anthropogenically mediated type of evolution, the phenotypic variation detected enables the artificial selection of genotypes that stand out in terms of productivity for subsequent recommendation in commercial planting aimed at increasing the agro-industrial productivity of sugarcane [25].

Our ANOVA results also revealed significant trait differences among the evaluated environments, thereby confirming the influence of edaphoclimatic conditions, such as temperature, relative humidity, amount and distribution of rainfall, light intensity, photoperiod, and soil moisture of the respective microregions, on genotypes [26]. Moreover, the significant interactions between genotypes and environments that we identified indicate the differential behavior of different genotypes in their respective microregions, which may be attributed to the differing amounts of rainfall in these microregions. Sugarcane requires 1,500–2,500 mm of rainfall that is well-distributed over the course of the growth cycle; these requirements are not met in all microregions in which genotypes are evaluated, and this is reflected in the phenotypic expression of certain traits [27]. This emphasizes the value of adaptability and stability analyses.

These results are similar to those obtained by Bastos *et al*. [28], who evaluated the agro-industrial performance of sugarcane genotypes in seven environments in the state of Minas Gerais, identified significant environmental interactions, and compared different methods of adaptability and stability analyses for more informed recommendations regarding genotypes. Similarly, Magalhães *et al*. [29] analyzed adaptability and stability and verified significant interactions of RB genotypes in two microregions over three harvest cycles. These results are important with respect to the adoption of varietal management. As enhancing environments and cultural practices is costly, it is possible to capitalize on the effects of genotype–environment interactions by distributing genotypes in environments in which they show the highest productivity.

Heritability coefficients showed high magnitude for the traits TPH, TCH, and TRS (t·ha^-1^) and average magnitude for the traits FIB, CP, PT, and TRS (kg), leading to significant gains in the selection of these materials with respect to the evaluated environments [30]. In addition, the high values obtained indicate a strong likelihood of the transmission of alleles responsible for the expression of these traits in hybridization studies. Thus, it is essential to evaluate genetic divergence to form families with better heterotic effects.

The traits TPH (t.ha-^1^), TCH (t.ha-^1^), and TRS (t.ha-^1^) also showed that the genotypic variance is superior to the non-genetic variances. This result demonstrates that the phenotypic expression of these traits is mostly due to the genetic potential of the genotypes evaluated in different environments.

With regard to the traits TPH, TCH, and TRS (t.ha^-1^), we obtained genotypic coefficient of variation values greater than 10, which, according to Oliveira *et al*. [31], are considered high. Moreover, Carvalho *et al*. [32] maintained that values of this magnitude are indicative of a high fraction of genotypic variance in the total phenotypic variation, indicating that selection should focus primarily on these traits. In this respect, our Scott–Knott grouping indicated that the genotypes RB92579, RB012777, RB863129, and SP79-1011 are potentially the most productive and cost-effective. Consistent with these considerations, Pimentel *et al*. [33] suggested that for a significant genetic gain, genotypes with high productivity should be selected. In the present study, based on the predicted genetic gains, we reasoned that not only RB92579 but also RB012777, RB863129, and SP79-1011 should be selected, given a degree of diversification, with different areas planted with distinct genotypes, thereby minimizing the potential risk associated with genetic uniformity, such as susceptibility to certain pathogens and other evolutionary forces [34].

Genetic gain for the traits FIB, CP, PT, SSC, and TRS (kg) tended to be somewhat less impressive, which is in line with expectations, given the average magnitudes of the values obtained for heritability coefficients. In this regard, Santos *et al*. [35] suggested that although the selection of superior genotypes associated with these traits is feasible, it tends to be costly.

According to Rosado *et al*. [36], selection based on the highest genotypic values of HMRPGV **×** GM implies the selection for adaptability, stability, and productivity. In the present study, among the assessed genotypes, we identified RB92579 as the most stable with respect to the traits TCH, TPH, and TRS (t·ha^-1^), followed by genotypes RB012777, RB863129, and SP79-1011. These four genotypes showed higher average productivity and gross economic profitability, higher independent genotypic averages, and higher values associated with adaptability and stability. Analyses of the genetic divergence among these genotypes should accordingly be carried out based on the genotypic values that combine the parameters of adaptability, stability, and high productivity.

Although the traits FIB, CP, PT, SSC, and TRS (kg) were characterized by a comparatively lower fraction of genetic variation, as expressed in terms of the coefficients of genotypic variation, they were stable in the genotypes that did not show high productivity. Some studies, including that by Morais *et al*. [37], maintained that such traits are qualitative in nature; that is, controlled by only few genes, which would explain the low genetic variation between genotypes and the relatively high stability in the assessed environments. However, we found that the heritability coefficient, which is normally high for qualitative traits, was not correspondingly high for these measured traits. Thus, we suggest that successive selection cycles for the improvement of these traits, which has the effect of promoting a narrowing of the genetic base, is a more plausible explanation for the low genetic variation among genotypes as well as for the high stability and difficulty in obtaining significant genetic gains. Reaching this conclusion was, however, only possible through the high informative power of mixed linear models, as the low values of the coefficients of determination confirmed the small contribution of non-genetic variance in the phenotypic variance of the genotypes.

Given that we detected genotypic correlations of medium to high magnitude with respect to the traits FIB, CP, PT, SSC, and TRS (kg), which were stable in genotypes of low productivity, we recommend using these genotypes in the analysis of genetic divergence. This is based on the possibility that during recombination, the effects of alleles responsible for the expression of these traits tend to manifest at the individual level and have a greater heterotic effect within the families obtained.

When the analyses of genetic divergence were performed using a traditional approach with the available phenotypic information for the evaluated traits, genotypes RB012776 and RB92579 were the most genetically distant (D^2^ = 21.63), and RB0122650 and RB012688 were the most similar (D^2^ = 0.50). In this type of analysis, a proportion of the environmental variance of traits is present to phenotypic variance. Similarly, the analysis of genetic divergence based on the genotypic values of adaptability and stability indicated that genotypes RB012776 and RB92579 were the most genetically distant (D^2^ = 24.18). In contrast, genotypes RB012633 and RB012688 were the most similar (D^2^ = 0.67).

The methodology proposed in this study can be used to evaluate genetic divergence using only the genotypic information of the evaluated traits, as mixed models enable extraction of the fraction of phenotypic variance attributable to environmental variance, thereby yielding unit or scale results for the evaluated trait. This approach encourages the careful consideration of hybridization planning in breeding programs.

In the analysis of genetic divergence based on phenotypic data, genotypes can be classified as more divergent or more genetically similar owing to the influence of the environmental variance that is present in the trait phenotypic variance and is computed in the analysis. The most divergent genotypes are identified using the trait genotypic values, because the environmental variance that disguises the true genetic potential of the trait is removed.

The moderate correlation between the matrices of genetic divergence was due to the traits being quantitative in nature. However, the number of genes that control their phenotypic expression varies considerably. This variation in the number of genes that control these traits can be visualized by analyzing the variation coefficients in several sugarcane improvement programs. It is more difficult to determine experimental accuracy for the traits TPH and TCH. The coefficients of variation for these traits usually have medium magnitude [38]. In contrast, the coefficients of variation for the traits FIB (%), CP (%), PT (%), SSC (%), and TRS (Kg) have low magnitude [37]. This indicates that quantitative traits controlled by a higher number of genes tend to be more influenced by environmental variations. The proportion of environmental variance is different in the most diverse quantitative traits.

We observed a moderate correlation between phenotypic and genotypic data. In the first group of data, although the traits showed environmental variance, in some, genetic variance predominated over non-genetic variances, as shown using analysis of genetic parameters (Table 5). Regarding the genotypic results, the influence of environmental variances was extrated using the mixed model methodology. Therefore, the correlation between the matrices was moderate, changing the genotype positions in the groups. Analyses of Genotypic data, for example, showed the formation of four groups (Table 10).

Vieira *et al*. [39], who evaluated the genetic divergence in wheat genotypes based on morphological (qualitative), phenological, and quantitative traits, verified a low correlation of distance matrices and attributed this low correlation to the different nature of the traits occupying distinct regions in the genome. If only quantitative traits had been evaluated, the correlation would have been higher.

The Tocher optimization method considers genotypes allocated to the same group, which thus tend to show high similarity with respect to the evaluated traits. Consequently, crossings should be carried out using genotypes allocated to different groups. Accordingly, taking into consideration divergence based on the phenotypic data, genotype 1 in the present study should be crossed with either genotype 9 or 10 only.

The divergence based on the genotypic values of adaptability and stability (HMRPGV × GM) showed that genotypes 2, 4, 6, and 8 belong to a different group compared with genotypes 1, 3, 5, and 7. Therefore, they were allocated to group II. Genotypes 9 and 10 showed similarity and were allocated to group III. Finally, genotype 11 was allocated alone to group IV.

This difference in the genotype allocation showed the accuracy of the analysis based on genotypic values. Considering the divergence based on phenotypic values and with the aim of obtaining new families with high adaptability and stability potential, genotype crossings should be made as follows: 2 × 9, 3 × 9, 5 × 9, 1 × 9, 11 × 9, 7 × 9, 8 × 9, 4 × 9, 6 × 9, 2 × 10, 3 × 10, 5 × 10, 1 × 10, 11 × 10, 7 × 10, 8 × 10, 4 × 10, 6 × 10, and 9 × 10, for a total of 19 crossings.

However, considering the divergence based on genotypic values, the following genotype crossings should be made: 1 × 2, 1 × 4, 1 × 6, 1 × 8, 3 × 2, 3 × 4, 3 × 6, 3 × 8, 5 × 2, 5 × 4, 5 × 6, 5 × 8, 7 × 2, 7 × 4, 7 × 6, 7 × 8, 1 × 9, 1 × 10, 1 × 11, 3 × 9, 3 × 10, 3 × 11, 5 × 9, 5 × 10, 5 × 11, 7 × 9, 7 × 10, 7 × 11, 2 × 9, 2 × 10, 2 × 11, 4 × 9, 4 × 10, 4 × 11, 6 × 9, 6 × 10, 6 × 11, 8 × 9, 8 × 10, 8 × 11, 9 × 11, and 10 × 11, for a total of 42 crossings.

In terms of efficiency, the present study analyzed 11 genotypes, and the reallocation of some of these genotypes to other groups caused considerable changes to crossing planning. The evaluation of a large number of genotypes can cause even greater changes to the identification of parents and definition of crosses.

In summary, genetic divergence is more efficient when based on genotypic values free from genotype–environment interactions, since the environmental effect can modify the phenotypic expression of the traits, compounding the analysis [40]. By crossing divergent genotypes based on genotypic data, the probability of allelic complementarity is expected to increase, that is, to concentrate alleles favorable to the greater manifestation of heterosis on the genotypes that will constitute the obtained families.

In addition to being simple, easy to use, and low cost, our methodology opens up new areas of study, for example, evaluating the parameters of adaptability and stability of a group of genotypes in intensive agricultural systems that use the most diverse technologies, such as irrigation with wastewater, vinasse, and optimization of fertilizer doses or even in rainfed environments. The genetic divergence of these materials that responded favorably to these conditions can be analyzed to obtain cultivars with higher productivity that are specifically adapted to certain conditions. Interestingly, there is no impediment for the respective analyses to be tested in other crops to maximize the accuracy in choosing the most useful parents for the genetic improvement of each crop.

## Supporting information

S1 Data (ZIP)
