# Correction: Genetic divergence for adaptability and stability in sugarcane: Proposal for a more accurate evaluation

**DOI:** 10.1371/journal.pone.0316561

**Published:** 2024-12-23

**Authors:** The PLOS ONE Staff

Table 3 is incorrect. It is a repeat of Table 4. Please see the correct Table 3 here. The publisher apologizes for the error.

**Table 3:** Summary of Joint Analysis of Variance of the Traits of Sugarcane Genotypes Evaluated Over Two Consecutive Years During the Final Phases of Trials Conducted in Sugarcane Microregions in the State of Pernambuco.

		Mean squares							
SV	DF	TPH (t.ha^-1^)	TCH (t.ha^-1^)	FIB (%)	CP (%)	PT (%)	SSC (%)	TRS (kg)	TRS/ (t.ha^-1^)
**Genotypes**	10	128.9**	5430**	7.22**	3.89*	48.2*	2.1*	189*	121**
**Years**	1	1362.2*	62785*	3.04^ns^	1.24^ns^	174^ns^	1.5^ns^	0.01^ns^	1348*
**Environments**	4	946.4**	36567**	53.2**	171**	813**	361**	13660**	846**
**G × Y**	10	8.7*	308.9*	1.13^ns^	1.37^ns^	5.9^ns^	2.2*	96^ns^	8.2*
**G × E**	40	13.49**	519**	2.12**	1.70**	22.3**	1.6**	96**	12.3**
**Y × E**	4	189.1**	6329**	17.5**	36.8**	78**	117**	3426**	188**
**G × Y × E**	40	3.79**	124.9*	1.15*	0.76^ns^	10.7*	1.0^ns^	51^ns^	3.46**
**Residue**	300	2.07	79.63	0.72	0.68	7.10	0.84	45	1.88
**Mean**		10.19	68.19	14.4	14.8	86.4	21.2	145.7	9.98
**CVe (%)**		14.14	13.08	5.89	5.56	3.0	4.32	4.6	13.7
**H**		4.0	4.4	4.20	3.0	7.0	2.9	2.7	3.7

**p = 0.05 and *p = 0.01 determined using an F test. ns, not significant; SV, source of variation; DF, degrees of freedom; G × Y, genotype × year interaction; G × E, genotype × environment interaction; Y × E, years × environment interaction; G × Y × E, genotype × year × environment interaction; CVe, coefficient of variation; H, Hartley test; TCH, tons of cane per hectare; TPH, tons of pol per hectare; FIB, fiber; CP, corrected pol%; PT, purity; SSC, soluble solids content; TRS, total recoverable sugars; and TRS/t·ha^-1^, total recoverable sugars per hectare.
